# Prevalence of chronic periodontitis in patients undergoing peritoneal dialysis and its correlation with peritoneal dialysis-related complications

**DOI:** 10.1186/s12882-023-03102-8

**Published:** 2023-03-24

**Authors:** Zhihao Chen, Hai Deng, Kristine Sun, Zehui Huang, Shan Wei, Yunyao Lin, Zhongchen Song, Yingli Liu

**Affiliations:** 1 Division of Nephrology, Shanghai Ninth People’s Hospital, School of Medicine, Shanghai Jiao Tong University, Shanghai, China; 2 Department of Periodontology, Shanghai Ninth People’s Hospital, School of Medicine, Shanghai Jiao Tong University School of Medicine, Shanghai, China; 3 National Clinical Research Center for Oral Diseases, Shanghai Key Laboratory of Stomatology & Shanghai Research Institute of Stomatology, Shanghai, China

**Keywords:** Peritoneal dialysis, Periodontitis, Peritonitis, Cardiovascular disease

## Abstract

**Objective:**

The microinflammatory state can influence the occurrence of dialysis-related complications in dialysis patients. Chronic periodontitis (CP), in which plaque biofilm is considered to be the initiating factor, is a chronic infectious disease in the oral cavity. It is still uncertain whether CP affects the microinflammatory state in peritoneal dialysis (PD) and the occurrence of dialysis-related complications. The purpose of this study was to investigate the correlation between the periodontal index and clinical parameters in peritoneal dialysis patients with CP and dialysis-related complications, including peritoneal dialysis-associated peritonitis (PDAP) and cardiovascular and cerebrovascular events (CCEs).

**Methods:**

This was a retrospective cohort study, and 76 patients undergoing PD were enrolled. Clinical parameters, the occurrence of PD-related complications and periodontitis-related indicators, including the gingival index (GI), plaque index (PLI), probing depth (PPD) and clinical attachment loss (CAL), were collected. Correlation analysis was used to explore the correlation between periodontal or clinical parameters and the occurrence of PD-related complications.

**Results:**

All the patients had different degrees of periodontitis (mild 9.2%, moderate 72.4%, severe 18.4%); PPD was inversely related to serum albumin (*r* = − 0.235, *p* = 0.041); CAL has a positive correlation with serum C-reactive protein (rs = 0.242, *p* = 0.035); PLI was positively correlated with serum calcium (*r* = 0.314, *p* = 0.006). ANOVA, multivariate logistic regression analysis and Kaplan-Meier Survival curve suggested that CAL was a risk factor for the occurrence of PDAP. There was no correlation between periodontal parameters and CCEs or poor prognosis.

**Conclusion:**

CP is universally present in PD patients, and the presentation of periodontitis influences the systemic inflammatory state in PD patients. CP is a risk factor for PDAP.

**Supplementary Information:**

The online version contains supplementary material available at 10.1186/s12882-023-03102-8.

## Introduction

Chronic kidney disease (CKD) affects approximately 10% of adults worldwide, leading to a large number of end-stage renal disease (ESRD) patients [1]. Peritoneal dialysis (PD), a type of renal replacement therapy, has the advantages of preserving residual renal function and mitigating cardiovascular risk; thus, it is a high-quality and cost-effective treatment for ESRD patients [2]. Current studies suggest that ESRD patients are in a state of continuous systemic microinflammation, and the levels of various inflammatory cytokines in blood circulation are continuously increasing [3]. Microinflammatory status is an important risk factor for cardiovascular events in dialysis patients [4, 5].

Chronic periodontitis (CP) is an inflammatory and destructive disease with multiple microbial sources and is characterized by gingival inflammation, periodontal attachment loss, alveolar bone resorption and periodontal pocket formation [6, 7]. The natural history of CP slowly progresses from the early gingivitis stage to irreversible severe periodontitis [7]. There is growing evidence that CP is associated with systemic diseases, including diabetes mellitus, cardiovascular and cerebrovascular events, pulmonary infectious disease and chronic kidney disease [6–8]. Some studies have shown that there is a bidirectional relationship between CKD and CP. As CKD progresses, patients show considerable susceptibility to CP, and patients with poor renal function usually have poor oral hygiene and a higher proportion of moderate or severe CP [9]. Similarly, CP, a nontraditional risk factor for CKD, can accelerate the progression of CKD, and periodontal therapy can significantly improve renal function [10–12]. However, high-quality clinical data on whether the presence of CP affects PD patients’ microinflammatory state and the occurrence of dialysis complications is still lacking.

Local inflammation of the periodontal tissue affects body systems by altering the levels of blood inflammatory mediators, increasing the probability of systemic inflammation in PD patients [7, 13]. Peritoneal dialysis-associated peritonitis (PDAP) is the most common clinical complication of PD and seriously affects peritoneal ultrafiltration and dialysis efficiency, and it is an important cause of the termination of PD and even death [14–17]. Cardio- and cerebrovascular events (CCEs) are the first and most important cause of death among PD patients [18]. PD-related complications seriously affect treatment efficacy and quality of life. A large number of studies have shown that age, sex, diabetes, hypoalbuminemia and long dialysis vintage are risk factors for PDAP [5, 19]. However, few studies have assessed the correlation between oral condition or periodontitis incidence and PD-related complications in PD patients.

This was a retrospective cohort study that enrolled 76 PD patients and aimed to analyze the correlation between periodontitis parameters and the clinical indexes of PD patients and whether CP is associated with the occurrence of PDAP or CCEs. It is hoped that the risk factors that affect the quality of life and survival rate of PD patients can be identified, leading to early intervention, thus reducing the incidence of PD-related complications, improving the quality of life of PD patients and improving their long-term survival rate.

## Methods

### Study population

This study included a convenience sample of 76 PD patients enrolled between January 2017 and January 2022 from Shanghai Ninth People’s Hospital attached to Shanghai Jiao Tong University School of Medicine. The inclusion criteria for PD patients were as follows:1) age 18–80 years and 2) regular stable PD for more than 3 months. The exclusion criteria were as follows:1) completely edentulous patients; 2) those with a history of any periodontal treatment in the past 3 months; 3) those who underwent conventional hemodialysis or renal transplantation; 4) those who received immunosuppressive therapy within the past 3 months; 5) those with uncontrolled systemic diseases like malignant tumors or acquired immune deficiency syndrome; and 6) nicotine or alcohol addiction. The end point of follow-up was as follows: 1) patients who converted to hemodialysis or underwent renal transplantation, and 2) death.

### Study methods

A full-time peritoneal dialysis nurse assumed responsibility for the patients’ medical records and blood sample database. A specific person took charge of the data entry and verification of the database. PD patients underwent full-mouth clinical periodontal examination by a trained and calibrated periodontist.

We collected all enrolled PD patients’ baseline characteristics (including age, sex, dialysis vintage, primary etiology and diabetes mellitus history DM), and patients were divided into a long PD vintage group (dialysis time ≥ 36 months, 47 patients) and a short PD vintage group (dialysis time < 36 months, 29 patients). The relevant laboratory data from the last PD adequacy assessment after January 2017 (including C-reactive protein (CRP), albumin (Alb), hemoglobin (Hb), calcium (Ca), phosphorus (P), and parathyroid hormone (PTH)) were collected. After professional periodontal examination, we recorded the occurrence of PDAP and CCEs. The PDAP diagnostic criteria were 1. clinical manifestations of peritonitis, including abdominal pain and cloudy peritoneal fluid; 2. dialysis effluent leukocyte count > 100/μl and neutrophils > 50%; and 3. positive bacterial culture results from dialysis effluent. A diagnosis could be made when the patient met at least two of the above criteria. CCEs included myocardial infarction, heart failure, acute coronary syndrome, cerebral infarction and cerebral hemorrhage.

The clinical evaluation was performed by UNC-15 periodontal probe (Hu-Friedy, Chicago, United States). Six sites (mesio-buccal, mid-buccal, distal-buccal, mesio-lingual, midlingual, and distal-lingual) per tooth were measured. Periodontal professional examination included the gingival index (GI), plaque index (PLI), probing depth (PPD), clinical attachment loss (CAL) and bleeding on probing (BOP).

According to the gingival color and tendency of bleeding on probing, the GI was used to evaluate gingival inflammation (most commonly, Löe and Silness, 0 to 3 scale). GI values were scored as: 0-normal gingiva; 1-mild inflammation (slight change in color, slight edema, no bleeding on probing); 2-moderate inflammation (redness, edema and glazing, bleeding on probing); 3-severe inflammation (marked redness and edema, ulceration, tendency to spontaneous bleeding). Based on the presence of bleeding after probing, BOP(+)% was calculated as the ratio of the number of bleeding sites to the number of examined sites, which is an objective indicator of inflammation. Patients with a mean percentage of sites with BOP (BOP%) of ≤20% are periodontally stable, while those with a mean BOP% of ≥30% are considered to have an increased risk of periodontal disease progression. PLI was used to evaluate oral hygiene based on dental plaque thickness. PLI values were evaluated as: 0-no bacterial plaque deposits; 1-the plaque deposit did not cover more than 1/3 of the coronary area; 2-the plaque deposit covered more than 1/3 but did not exceed 2/3 of the coronary area; 3-the plaque covered more than 2/3 of the dental surface. GI, PLI and BOP% could represent the oral hygiene and gingival condition, not the definition of periodontitis, but they are still the essential periodontal examinations.

The diagnosis of Periodontitis is mainly determined by CAL. According to the classification criteria for periodontal diseases, gingival inflammation and bleeding on probing, PPD ≤ 4 mm, and 1 mm ≤ CAL ≤ 2 mm was defined as mild CP; gingival inflammation and bleeding on probing, 4 mm < PPD ≤ 6 mm, 3 mm ≤ CAL ≤ 4 mm, and a probable loose tooth was defined as moderate CP; PPD>6 mm, CAL ≥ 5 mm, periodontal lesions including furcation involvement, and significant inflammation or periodontal abscess was defined as severe CP. When CAL was between 2 and 3 mm or 4–5 mm, the degree of periodontitis was determined by PPD and periodontal symptoms [20].

Grouping was based on the severity of CP and the presence of adverse events after periodontal examination. According to the severity of CP, patients were divided into mild group (7 patients), moderate group (55 patients) and severe group (14 patients). According to the presence or absence of PDAP, patients were divided into a PDAP group (33 patients) and a non-PDAP group (43 patients); similarly, patients were divided into a CCE group (26 patients) and a non-CCE group (50 patients).

All patients did not receive the periodontal therapy.

### Statistical analyses

All data were analyzed using SPSS software (version 23.0;IBM Corporation)for Microsoft Windows. The normally distributed variables are described using the mean and standard deviation (SD), while the median and interquartile range (IQR) are used to describe nonnormally distributed data, and frequency (percentage) is used to describe qualitative data. Statistical significance was determined using independent samples t tests when the data were normally distributed. Multigroup comparisons were performed using ANOVA. The Wilcoxon rank-sum test was used if the results consisted of nonnormally distributed data or ordinal data. The chi-squared test was used for categorical variables. Correlation analysis was performed by Spearman’s or Pearson’s correlation method for nonnormally or normally distributed data. Binary logistic regression models were constructed to investigate risk factors. Survival curves were constructed by the Kaplan-Meier method. A *p* value < 0.05 was considered to indicate statistical significance.

## Results

### Baseline characteristics and clinical parameters

A total of 76 patients (45 males and 31 females, mean age: 60.37 years) were included in this study. As shown in Table 1, all patients had different degrees of periodontitis, and the proportion of patients with moderate or severe periodontitis reached 90.8%. Table 1 also shows other clinical parameters and periodontal indexes in PD patients.

**Table 1:** Basic characteristics and clinical parameters

Index	Total(*n* = 76)
Age(years)	60.37 ± 14.35
Gender(M/F)	45/31
**Primary etiology**	
Diabetic nephropathy	23(30.7%)
Hypertensive nephropathy	12(15.8%)
Chronic glomerulonephritis	17(22.4%)
Polycystic kidney	2(2.6%)
Systemic lupus erythematosus	1(1.3%)
Multiple myeloma	1(1.3%)
Pyelonephritis	1(1.3%)
Unknown	19(25.0%)
**Comorbidities**/n(%)	
DM	33(43.4%)
CHD	62(81.6%)
**PD vintage**/n(%)	
3–36 months	29(38.2%)
>36 months	47(61.8%)
Alb(g/L)	33.26 ± 5.00
Hb(g/L)	102.54 ± 20.61
Ca(mmol/L)	2.24 ± 0.27
P(mmol/L)	1.80 ± 0.55
PTH(pg/mL)	243.18(149.20,480.43)
ALP(U/L)	88.00(72.00,146.75)
CRP(mg/L)	3.34(0.79, 9.20)
**Periodontal parameters**	
PPD(mm)	3.66 ± 0.94
CAL(mm)	3.91 ± 1.21
GI	1.84(1.73, 2.00)
PLI	1.82 ± 0.43
BOP(+)%	81.98(72.22, 90.96)
**Degrees of CP**/n(%)	
mild	7(9.2%)
moderate	55(72.4%)
severe	14(18.4%)

*M/F* Male/female, *DM* Diabetes, *CHD* Coronary heart disease, *PD* Peritoneal dialysis, *CP* Chronic periodontitis, *PPD* Probing depth, *CAL* Clinical attachment loss, *GI* Gingival index, *PLI* Plaque index, *BOP* Bleeding on probing

Tables 2 and 3 show the incident rate of PDAP and CCEs. During 208.7 patient-years, 26 patients developed 32 episodes of CCEs and the incident rate of CCEs was 0.15 episodes/patient-year. During 229.3 patient-years, 33 patients developed 44 episodes of peritonitis and the incident rate of peritonitis was 0.19 episodes/patient-year.

**Table 2:** Basic characteristics and the data on CCE

	CCE(*n* = 26)
**Episodes/patients-years**	0.15
**Classification of Disease**	
Heart failure	13(40.6%)
Acute Coronary Syndrome	5(15.6%)
Infarct of Brain	12(37.5%)
Hematencephalon	2(6.3%)

*CCEs* Cardiovascular and cerebrovascular events

**Table 3:** Basic characteristics and the data on PDAP

	PDAP(*n* = 33)
**Episodes/patients-years**	0.19
**Bacteria**	
Gram-positive cultures	26(59.1%)
Staphylococcus species	12(27.3%)
Streptococcus species	10(22.7%)
Others	4(8.7%)
Gram-negative cultures	10(22.7%)
Culture-negative	8(18.2%)

*PDAP* Peritoneal dialysis-associated peritonitis

### The correlation between clinical parameters and periodontal indexes

As shown in Table 4, PPD was weakly correlated with Alb (*r* = − 0.235, *p* = 0.041). There were positive correlations of CAL with CRP and of PLI with Ca (rs = 0.242, *p* = 0.035; *r* = 0.314, *p* = 0.006). The correlations between the remaining clinical and periodontal parameters were not statistically significant.

**Table 4:** Pearson correlation coefficient(r) or Spearman’s rank correlation coefficient(r_s_) between PD clinical parameters and indices of CP

	PPD(mm)	CAL(mm)	PLI	GI(r_s_)	BOP(+)%(r_s_)
Alb(g/L)	−0.235^①^	− 0.165	− 0.016	−0.016	− 0.047
Hb(g/L)	0.166	−0.067	0.140	−0.073	0.007
Ca(mmol/L)	−0.015	−0.053	0.314^③^	0.158	0.130
P(mmol/L)	−0.158	−0.070	− 0.142	0.076	− 0.067
PTH(pg/mL, r_s_)	−0.184	− 0.086	−0.105	− 0.053	−0.057
ALP(U/L, r_s_)	0.020	0.062	0.083	0.029	0.052
CRP(mg/L, r_s_)	0.137	0.242^②^	0.086	0.138	0.054

*PPD* Probing depth, *CAL* Clinical attachment loss, *GI* Gingival index, *PLI* Plaque index, *BOP* Bleeding on probing

①:*p* = 0.041;②:*p* = 0.035;③:*p* = 0.006

### Differential analysis with ANOVA

As shown in Table 5, patients’ characteristic were compared between patients with mild, moderate and severe CP. Long PD vintage (*p* = 0.029), CRP (*p* < 0.01) and the occurrence of PDAP (*p* < 0.01) were significantly different between patients with the different severity of CP.

**Table 5:** ANOVA test of the different severity of CP

	Mild (*n* = 7)	Moderate (*n* = 55)	Severe (*n* = 14)	χ^2^/H	*P* value
Age(years)	61.71 ± 15.88	59.04 ± 15.00	64.93 ± 10.30	0.974	0.382
Gender(M/F)	5/2	31/22	9/5	0.372	0.697
Long PD Vintage	6(85.71%)	29(52.73%)	12(85.71%)	3.707	0.029
DM	4(57.14%)	21(38.18%)	8(57.14%)	1.100	0.338
Alb(g/L)	36.71 ± 3.90	33.11 ± 5.15	32.14 ± 4.40	2.102	0.130
Hb(g/L)	105.14 ± 27.16	102.75 ± 20.56	100.43 ± 18.60	0.129	0.879
Ca(mmol/L)	2.40 ± 0.49	2.20 ± 0.22	2.31 ± 0.29	2.496	0.089
P(mmol/L)	1.59 ± 0.45	1.86 ± 0.59	1.67 ± 0.43	1.232	0.298
PTH(pg/mL)	238.05(46.98,456.80)	252.60(167.50,525.99)	150.57(95.19,374.48)	0.766	0.468
ALP(U/L)	71.00(63.00,138.00)	92.00(77.00,146.00)	86.00(67.25,151.75)	0.373	0.690
CRP(mg/L)	1.68(0.00,4.26)	2.43(0.00,47.56)	18.25(3.00,40.14)	16.107	< 0.001
The occurrence of complications					
CCEs	4(57.14%)	18(32.73%)	4(28.57%)	0.929	0.399
PDAP	0(0.00%)	21(38.18%)	12(85.71%)	9.872	< 0.001

*CP* Chronic periodontitis, *M/F* Male/female, *DM* Diabetes, *CCEs* Cardio- and cerebrovascular events, *PDAP* Peritoneal dialysis-associated peritonitis

### Single-factor analysis in patients with peritoneal dialysis-related complications

There were no significant differences in any periodontal indexes (PPD, CAL, PLI, GI, BOP+%) between patients with or without CCEs (*p* = 0.948, *p* = 0.616, *p* = 0.431, *p* = 0.473, *p* = 0.974) (Table S.1).

As shown in Table S.2, the values of CP (z = − 3.992, *p* < 0.001), CRP (z = − 2.582, *p* = 0.01) and long dialysis vintage (χ^2^ = 5.663, *p* = 0.017) were significantly different between patients with and those without PDAP. The differences between other variables and the occurrence of PDAP were not statistically significant.

### Multivariate logistic regression analysis of risk factors affecting the occurrence of PDAP

As shown in Table 6, we use three multivariate-adjusted models to evaluate the association of CP and the occurrence of PDAP. In multivariate-adjusted model 1, dialysis vintage, CRP and periodontal parameters (PPD and PLI) were adjusted in the binary logistic regression analysis (all variables were significantly different between patients with and those without PDAP in single-factor analysis). In model 2, we adjusted DM and Alb, which are known to associate with a higher risk of peritonitis, but show no significant differences in this study. In model 3, we adjusted for potential confounders (age and sex). After adjusting for all the variables, Table 6 shows that the occurrence of PDAP were significantly associated with increasing CAL.

**Table 6:** Multivariate logistic regression analysis of risk factors affecting the occurrence of PDAP

Variable	Multivariate-Adjusted Model 1		Multivariate-Adjusted Model 2		Multivariate-Adjusted Model 3	
	OR(95% CI)	*P* value	OR(95% CI)	*P* value	OR(95% CI)	*P* value
CAL(mm)	2.135(1.094–4.168)	0.026	2.107(1.056–4.205)	0.034	2.159(1.047–4.455)	0.037

Adjusted 1 model adjust for: PPD; PLI; CRP; Long PD vintage. Adjusted 2 model adjust for:PPD; PLI; CRP; Long PD vintage; Alb; DM; Adjusted 3 model adjust for: Age; Sex; PPD; PLI; CRP; Long PD vintage; Alb; DM

### Kaplan-Meier survival curve for the occurrence of PDAP

**Fig. 1:**
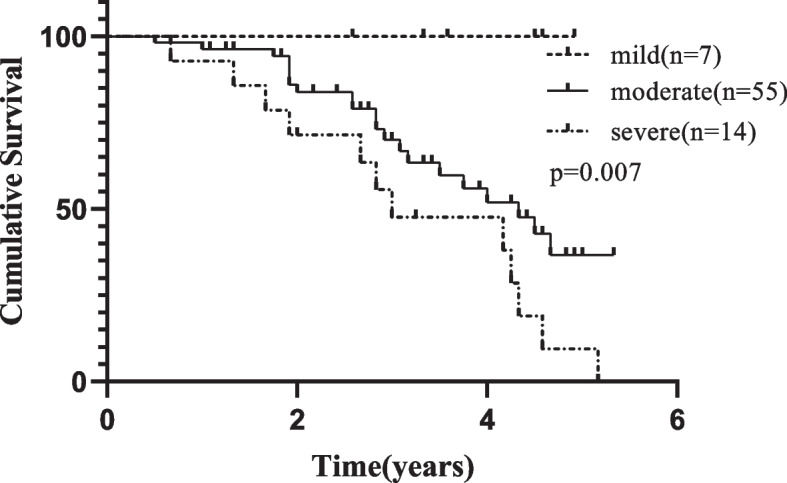
Kaplan-Meier Survival Curve for the occurrence of PDAP

In order to reduce the lead-time bias, the risk factor of PDAP was estimated using the Kaplan-Meier method. The result was presented in Fig. 1. It revealed that the severity of CP is the risk factor of PDAP (*p* = 0.007).

## Discussion

There are many common risk factors for the occurrence and progression of CP and CKD (including age and environmental factors). The risk of the occurrence of severe periodontal disease increases as CKD progresses to ESRD [12]. There are many reasons for susceptibility to periodontal disease in ESRD patients. To prevent overload volume,dialysis patients need to restrict fluid intake and use diuretics as adjuvant therapy, especially patients with residual renal function; thus, xerostomia becomes the most common oral symptom [7, 21]. On the other hand, uremia alters the inflammatory response to bacterial plaques in gingival tissue, and reduced salivation can further lead to plaque formation [7]. Currently, the proportion of regular stomatology follow-up in ESRD patients is very low [9]. Negligence of oral hygiene is one of the main causes of a higher prevalence and severity of CP [7, 22, 23]. Numerous studies have shown that the prevalence of moderate or severe periodontitis in PD patients was significantly higher than that in those without CKD, and CP was widespread in patients with renal replacement therapy. Patients with CKD stage 3 or higher also had a significantly increased prevalence of CP [7, 12, 22, 24].

Similar results were obtained in this study. Based on the collected periodontal clinical data, patients’ GI, PLI and BOP(+)% were high, indicating poor oral hygiene and gingival status. PPD and CAL are the most important parameters for periodontitis. PPD is closely related to subgingival plaque biofilms and inflammatory status, representing the current CP condition. CAL is an indicator of previous cumulative tissue destruction [7]. Our study found that all PD patients had different degrees of CP(mild: 9.2%, moderate: 72.4%, severe 18.4%), and more than 90% of patients had moderate/severe CP. The mean value of CAL reached 3.91 mm. Ibrahim et al. [7] reported that the mean CAL reached 3.97 mm in predialysis patients. Kristine Sun et al. [24] found that there were significant differences in CAL between PD patients and healthy patients, indicating more severe periodontitis in PD patients. In a previous study of serological examination in dialysis patients, the serum CRP levels in hemodialysis patients with CP were significantly higher than those without CP. The serum CRP levels significantly decreased after periodontal treatment [25, 26]. Kristine Sun et al. [24] showed that various inflammatory factors (including IL-6, IL-8, hs-CRP, etc.) in gingival crevicular fluid were correlated with the degree of periodontitis and were much higher than those in healthy people. This phenomenon was related to periodontal tissue destruction caused by severe periodontal disease in PD patients. This study found a positive correlation between CRP and CAL, the serum CRP levels significantly positive correlated with the CP severity increasing in dialysis patients, indicating that periodontitis was probably an important source of systemic inflammation in PD patients. This is in keeping with the result of previous study [27].

The physiological function and metabolism were mediated with the prolonging of the dialysis time. It is interesting that duration of PD is significant correlated with the severity of CP in our study. Bayraktar G et al. [27] showed that GI and PPD scores were significantly higher in subgroups receiving HD for 3 or more years, and were positively correlated with HD duration. Several reports have indicated periodontal disease-related parameters, such as PPD, CAL, GI, and PLI, worsed with longer duration of HD [28, 29], but the relationship between the degree of periodontitis and dialysis vintage remains controversial.

Hypoalbuminemia not only is an indicator of malnutrition but also is related to clinical complications, influencing the prognosis [30, 31]. This study showed a weak negative correlation between PPD and Alb (*r* = − 0.235, *p* = 0.041), probably because persistent inflammatory reactions and reduced oral intake are associated with malnutrition [9, 24, 26]. Periodontal status can affect nutritional parameters, and periodontal treatment can improve periodontal status as well as inflammatory status and the nutritional state [19]. However, some studies found no statistically significant association between Alb and the severity of periodontitis. In addition to nutritional status, these studies suggested that Alb is regulated by other factors, such as protein loss caused by peritoneal dialysis and gastric anorexia caused by inadequate dialysis [26, 31]. In our study, we just collect patients’ clinical parameter-Alb. More reliable nutritional markers, such as anthropometric assessment, body composition, other biochemical parameters (transthyretin, cholesterol and total lymphocytes, etc.) are needed to evaluate patients’ nutritional status [32]. Increased calcium in the saliva can promote dental calculus formation [7], which may be supported by the positive correlation between PLI and serum Ca in this study. On the other hand, secondary hyperparathyroidism can alter the levels of serum calcium and phosphorus metabolism and PTH, which are common complications observed in dialysis patients [22]. Kristine Sun et al. [24] and Naghsh N et al. [31] reported a positive correlation between PTH and alveolar bone loss and suggested that CKD-mineral and bone disorder (MBD) was an important risk factor for alveolar bone loss. Furthermore, alveolar bone loss can be improved by increasing calcium intake and worsened by a high-phosphorus diet [12, 33]. This study found only a weak correlation between Ca and PPD, while several studies showed that there were no statistically significant differences in Ca, P and PTH between PD patients with or without CP [26, 31, 33]. Interestingly, completely edentulous are common in PD patients. Although these patients were ineligible in this study, it may indicate cross-talk between CKD and CP. Therefore, we need further studies to prove that periodontitis is correlated with secondary hyperparathyroidism and calcium and phosphorus metabolism. Hematopoietic raw material, nutritional status and inflammatory states result in anemia in dialysis patients. This study showed no significant correlation between serum Hb and periodontitis, which is consistent with the results of most recent studies [31].

The presence of microinflammatory status in dialysis can lead to an increased risk for cardiovascular and infectious disease, seriously affecting the quality of life and prognosis [2, 7]. Some studies have suggested that the degree of CP is positively related to inflammatory parameters (including hs-CRP, etc.) and atherosclerosis risk factors [20, 25]. The relationship between periodontitis and systemic inflammation includes the cytokine response in the gingival epithelium caused by bacterial plaque, bacteremia caused by oral bacteria, circulating oral microbial toxins and immune responses to oral microorganisms [23]. Bacteremia and the systemic inflammatory response associated with CP are not only initiating factors for vascular endothelial lesions but also important factors of the vascular wall inflammatory process [20, 21]. Patients with CKD and CP as comorbidities can experience the progression of renal and cardiovascular disease due to systemic chronic inflammation [12]. This study also found a correlation between CAL and CRP. However, there were no significant differences in any periodontal parameters between patients with and those without CCEs. And the occurrence of CCEs did not differ significantly in the different severity PD patients. Tasdemir Z et al. [34] reported that periodontal therapy can reduce the systemic inflammatory response, thus contributing to a reduction in the risk of cardiovascular and cerebrovascular disease. However, evidence that CP determines the long-term effects on CCEs is still limited, and there is little evidence that periodontal treatment can prevent atherosclerosis or change its prognosis [20, 35]. Further intervention studies are needed to confirm the relationship between CP and CCEs in dialysis patients.

Currently, there are few studies on the correlation between oral infection and dialysis-related infection. Research on dialysis-related infections mostly focuses on the analysis of infectious pathogens and lacks research data on oral health conditions and oral microorganisms. No studies have reported the association between periodontitis and PDAP. ISPD guidelines [36] reported that oral streptococci can cause PDAP. Hideaki Oka et al. [23] suggested that better oral hygiene habits were associated with a lower incidence of PDAP and streptococcal infection. At present, the risk factors for PDAP are considered to include age, hypoalbuminemia, DM, etc. [19]. Moreover, a long dialysis vintage can aggravate periodontal damage [24]. In this study, all variables were included in the univariate analysis, and we found statistically significant differences in CAL, PPD, PLI, CRP, long PD vintage and the severity of CP between the occurrences of PDAP and non-PDAP. Then, these statistically significant variables were included in the multivariate regression analysis, we found that the severity of CP is significantly associated with the occurrence of PDAP, and this result was still significant after adjusting for confounding variables such as age, sex, Alb and the history of diabetes mellitus.

Bacteremia caused by invasive dental manipulation is considered one of the secondary causes of PDAP [23]. Oka H et al. [23] reported that oral streptococci can be found in PD effluent from some PDAP patients, and streptococci in the oral cavity was the most important bacterial cause of PDAP in the oral cavity. The study also summarized other previous studies to prove the theory that oral splash contamination leads to PDAP. To prevent exogenous peritonitis, the study recommended hand washing and wearing a face mask before fluid exchange. At the same time, the positive culture rate of gram-negative Enterobacteriaceae was 1.2–3.2% on the tongue and in saliva and gingival crevicular fluid, indicating that gram-negative Enterobacteriaceae derives from the gastrointestinal tract but also comes from the oral cavity as the main pathogenic bacteria. However, the main causal organisms of PDAP are still gram-positive bacteria, and staphylococci, as the most common bacteria found in PDAP patients, are not oral colonization bacteria [17]. In our study, gram-positive bacteria were the predominant pathogens (57.8%), with staphylococci and streptococci each accounting for 24.4% of infections. Unfortunately, we were not able to detect the presence of oral flora, so the relationship between oral pathogens and PDAP pathogens requires further study.

In addition, periodontitis can lead to poor glycemic control, consequently increasing the risk of other complications of DM [20]. After treatment, the inflammatory markers in PD patients, especially those with DM, are slightly reduced, and blood glucose control also improves [7, 20]. These results suggest that periodontal disease is the main source of inflammation in PD patients with DM. Due to the small sample of DM patients, the daily glucose levels of patients during follow-up were not accurately recorded, so data on the correlation between periodontitis and glycemic control were not available.

The main limitation of this study is that this is an observational cohort study with no periodontal therapy intervention, a lack of long-term control, a small sample size, and a short follow-up duration. It is just a convenience sample, this conclusion does not apply to all PD patients. Although our study found that CP can influence systemic inflammatory status, which is a risk factor for PDAP, it is not certain whether the improvement in periodontitis reduces systemic inflammation, improves nutritional status, slows the process of alveolar bone loss and reduces the incidence of PDAP and CCEs. The answers to these questions need to be confirmed by further prospective randomized controlled studies.

## Conclusion

In summary, chronic periodontitis is prevalent among peritoneal dialysis patients. The presence of periodontitis, which affects the inflammatory status, is a risk factor for PDAP. Additional study is needed to verify the correlation between periodontitis and nutritional condition or calcium-phosphorus metabolism. Therefore, clinicians need to pay attention to PD patients’ oral hygiene in their work, intervene in a timely manner, and treat periodontitis. After the improvement of oral hygiene, the patients’ immune defense ability and nutritional status may be improved. Further investigation needs to be performed in this area. To a certain extent, it is beneficial to prevent the occurrence of PDAP, improve the quality of life and reduce the occurrence of dialysis complications.

## Supplementary Information

**Additional file 1: Table S.1.** Comparison of the periodontal clinical parameters and the occurrence of cardiovascular complications.

**Additional file 2: Table S.2.** Univariate analysis of risk factors for PDAP.

## Data Availability

All data generated or analysed during this study are included in this published article and its supplementary information files.
