# Supplementary material for: Prevalence of chronic periodontitis in patients undergoing peritoneal dialysis and its correlation with peritoneal dialysis-related complications
Source: BMC Nephrol. 2023 Mar 24;24:71. doi: 10.1186/s12882-023-03102-8 (PMC10039550; doi:10.1186/s12882-023-03102-8)
Supplement: Supplementary file 1 — Additional file 1: Table S.1. Comparison of the periodontal clinical parameters and the occurrence of cardiovascular complications. [file 12882_2023_3102_MOESM1_ESM.docx]

Table S.1. Comparison of the periodontal clinical parameters and the occurrence of cardiovascular complications

|  | CCE group(n=26) | No CCE group(n=50) | t/Z  value | P  value |
| --- | --- | --- | --- | --- |
| PPD(mm) | 3.65±1.13 | 3.67±0.84 | -0.066 | 0.948 |
| CAL(mm) | 3.81±1.09 | 3.96±1.27 | -0.504 | 0.616 |
| PLI | 1.77±0.47 | 1.85±0.41 | -0.792 | 0.431 |
| GI | 1.91(1.72,2.04) | 1.84(1.74,1.97) | -0.717 | 0.473 |
| BOP(+)% | 79.78(69.39,92.42) | 82.74(72.30,90.12) | -0.033 | 0.974 |
| Degrees of CP/n(%) |  |  |  |  |
| mild | 4(15.4%) | 3(6.0%) |  |  |
| moderate | 18(69.2%) | 37(74.0%) | -1.076 | 0.282 |
| severe | 4(15.4%) | 10(20.0%) |  |  |

Note: CCEs:Cardio- and cerebrovascular events;
