# Supplementary material for: Prevalence of chronic periodontitis in patients undergoing peritoneal dialysis and its correlation with peritoneal dialysis-related complications
Source: BMC Nephrol. 2023 Mar 24;24:71. doi: 10.1186/s12882-023-03102-8 (PMC10039550; doi:10.1186/s12882-023-03102-8)
Supplement: Supplementary file 2 — Additional file 2: Table S.2. Univariate analysis of risk factors for PDAP. [file 12882_2023_3102_MOESM2_ESM.docx]

Table S.2. Univariate analysis of risk factors for PDAP

|  | PDAP group(n=33) | No PDAP group(n=43) | t/Z /χ² | p-value |
| --- | --- | --- | --- | --- |
| PPD(mm) | 4.05±0.96 | 3.37±0.82 | 3.303 | 0.001 |
| CAL(mm) | 4.53±1.13 | 3.44±1.05 | 4.346 | ＜0.001 |
| PLI | 2.00(1.54,2.21) | 1.79(1.38,2.04) | -2.002 | 0.045 |
| GI | 1.90(1.75,2.11) | 1.82(1.73,1.95) | -1.054 | 0.292 |
| BOP(+)% | 83.93(63.28,92.49) | 79.63(72.33,88.00) | -0.587 | 0.557 |
| Degrees of CP/n(%) |  |  |  |  |
| mild | 0(0.0%) | 7(16.3%) |  |  |
| moderate | 21(63.6%) | 34(79.1%) | -3.992 | ＜0.001 |
| severe | 12(36.4%) | 2(4.7%) |  |  |
| Age | 62.76±14.64 | 58.53±14.01 | 1.277 | 0.206 |
| Gender(Male)/n(%) | 19(57.6%) | 26(60.5%) | 0.592 | 0.442 |
| DM/n(%) | 16(48.5%) | 19(57.6%) | 0.609 | 0.435 |
| Long PD age | 25(75,8%) | 22(51.2%) | 5.663 | 0.017 |
| Alb(g/L) | 32.10±5.17 | 34.15±4.74 | -1.794 | 0.077 |
| CRP(mg/L) | 5.43(1.82,18.25) | 2.08(0.00,5.34) | -2.582 | 0.01 |

Note:PDAP:peritoneal dialysis-associated peritonitis
